# PERK regulates G_q_ protein-coupled intracellular Ca^2+^ dynamics in primary cortical neurons

**DOI:** 10.1186/s13041-016-0268-5

**Published:** 2016-10-01

**Authors:** Siying Zhu, Barbara C. McGrath, Yuting Bai, Xin Tang, Douglas R. Cavener

**Affiliations:** 1 Department of Biology, Center of Cellular Dynamics, the Pennsylvania State University, University Park, PA 16802 USA; 2 Whitehead Institute for Biomedical Research, 9 Cambridge Center, Cambridge, MA 02142 USA

**Keywords:** PERK, G_q_ protein-coupled receptor, Ca^2+^, Receptor-operated Ca^2+^ entry

## Abstract

PERK (EIF2AK3) is an ER-resident eIF2α kinase required for behavioral flexibility and metabotropic glutamate receptor-dependent long-term depression via its translational control. Motivated by the recent discoveries that PERK regulates Ca^2+^ dynamics in insulin-secreting β-cells underlying glucose-stimulated insulin secretion, and modulates Ca^2+^ signals-dependent working memory, we explored the role of PERK in regulating G_q_ protein-coupled Ca^2+^ dynamics in pyramidal neurons. We found that acute PERK inhibition by the use of a highly specific PERK inhibitor reduced the intracellular Ca^2+^ rise stimulated by the activation of acetylcholine, metabotropic glutamate and bradykinin-2 receptors in primary cortical neurons. More specifically, acute PERK inhibition increased IP_3_ receptor mediated ER Ca^2+^ release, but decreased receptor-operated extracellular Ca^2+^ influx. Impaired G_q_ protein-coupled intracellular Ca^2+^ rise was also observed in genetic *Perk* knockout neurons. Taken together, our findings reveal a novel role of PERK in neurons, which is eIF2α-independent, and suggest that the impaired working memory in forebrain-specific *Perk* knockout mice may stem from altered G_q_ protein-coupled intracellular Ca^2+^ dynamics in cortical pyramidal neurons.

**Electronic supplementary material:**

The online version of this article (doi:10.1186/s13041-016-0268-5) contains supplementary material, which is available to authorized users.

## Introduction

Calcium (Ca^2+^) serves as an important second messenger in the central nervous system, as it regulates various neuronal processes including neurotransmitter release, synaptic plasticity, neuron excitability, and neuronal gene transcription [1]. Initiators of intracellular Ca^2+^ rise in neurons include the G_q_-protein coupled receptors, whose activation upon agonist binding leads to the activation of G_q_/phospholipase C (PLC) pathway. Activated PLC hydrolyzes phosphatidylinositol 4,5-bisphosphate (PIP_2_) resulting in the generation of inositol 1,4,5-triphosphate (IP_3_) and diacylglycerol (DAG). While the increased cytosol IP_3_ induces internal Ca^2+^ release by binding with ER resident inositol-1,4,5-triphosphate receptor (IP_3_R), the activation of G_q_/PLC cascade further stimulates receptor-operated Ca^2+^ influx from external space.

The central nervous system expresses a variety of G_q_ protein-coupled receptors including the M1 and M3 muscarinic acetylcholine receptors (mAChR), the group 1 metabotropic glutamate receptor (mGluR1), and the bradykinin-2 receptor [2, 3]. G_q_ protein-coupled intracellular Ca^2+^ dynamics has been shown to play prominent roles in a number of neuronal processes. For example, in pyramidal neurons, G_q_ protein-coupled intracellular Ca^2+^ rise induced by mAChR or mGluR1 activation is required for the induction of Ca^2+^-activated nonselective cationic current (I_CAN_) [4, 5], which is considered the ionic mechanism underlying persistent neuronal firing essential for working memory. In cerebellar Purkinje cells, mGluR1-induced postsynaptic Ca^2+^ rise in dendritic spines has been shown to be essential for long-term synaptic depression [6].

PERK, an eIF2α kinase well known for its role in eIF2α-dependent protein synthesis and translational control, has been shown to modulate Ca^2+^ dynamics-dependent working memory [7], in addition to protein synthesis-dependent cognitive functions including learning and memory flexibility [8], and long-term depression mediated by mGluR1 [9]. Moreover, it has been demonstrated that PERK acutely regulates Ca^2+^ dynamics in β-cells underlying glucose-stimulated insulin secretion, which is independent on protein synthesis [10]. Considering the critical role of Ca^2+^ signaling in working memory, we hypothesized that PERK may also regulate Ca^2+^ dynamics in pyramidal neurons. We show herein that acute PERK inhibition impairs G_q_ protein-coupled intracellular Ca^2+^ rise. More specifically, PERK inhibition enhances IP_3_R mediated ER Ca^2+^ release, but suppresses receptor-operated external Ca^2+^ influx. G_q_ protein-coupled intracellular Ca^2+^ rise is also impaired in genetic *Perk* knockout neurons. Taken together, our findings suggest that PERK regulates G_q_ protein-coupled Ca^2+^ dynamics in pyramidal neurons, which may serve as the cellular mechanism underlying impaired working memory in forebrain-specific *Perk* knockout mice.

## Methods

### Reagents

PERK inhibitor GSK2606414 was a kind gift from Jeffery M. Axten and Rakesh Kumar, GlaxoSmithKline, Collegeville, PA. (S)-3,5-Dihydroxyphenylglycine (DHPG) and thapsigargin were purchased from Tocris, bradykinin acetate salt was purchased from Sigma, carbachol was purchased from EMD Millipore, and Bt_3_-Ins(1, 4, 5)P_3_/AM (IP_3_-AM) was purchased from SiChem.

### Primary neuron culture

Wild-type primary cortical neurons were prepared from wild-type mice colony in C57BL/6 J background as previously described [7]. Briefly, the cerebral cortex was isolated from day 0 pups and dissociated in 0.05 % trypsin-EDTA with DNase1 for 30 min at 37 °C (5 % CO^2^), followed by two washes with HBSS containing 10 % FBS and trituration in neuronal medium with DNase1. Dissociated cells were collected by centrifugation at 120Xg for 5 min and plated on glial-coated 12 mm glass coverslips in a 24-well plate at a density of approximately 150,000 cells per well. Total medium was changed on the 1^st^ day in vitro (DIV) and 50 % of the medium was changed on DIV 3 and DIV 5. Genetic *Perk* knockout neurons were prepared from *Perk-floxed Nestin-Cre* mice in the same way, and the genotype of each pup was determined later. The cells were maintained in MEM based neuronal medium containing 5 % FBS (GEMINI Bio-Products), 2 % B27 (Invitrogen), 1 mM L-Glutamine (Gibco), 20 mM D-Glucose, 2 μM Cytosine Arabinoside (AraC), 40 units/ml penicillin, 40 μg/ml streptomycin and 100 ng/ml Amphotericin B, and final pH was adjusted to 7.4 with NaHCO_3_ (100 mg/500 ml). DIV 14–19 neurons were used for Ca^2+^ imaging experiments and immunocytochemistry.

### Intracellular Ca^2+^ measurements and Ca^2+^ imaging data analysis

Intracellular Ca^2+^ levels were measured using the ratiometric Ca^2+^ probe Fura-2 AM (Molecular Probes). Briefly, coverslips seeded with neurons at DIV 14–19 were incubated in bath solution with 2 μM Fura-2-AM for 30 min at room temperature in the dark. Coverslips were then transferred to fresh bath for 15 min to allow the cleavage of AM esters by cellular esterases. After dye loading, the coverslips were put in a perfusion chamber mounted on Nikon TE-200-S inverted microscope with Xenon arc lamp as the fluorescence excitation source. Ratios of images with the fluorescent emission signal excited at 340 nm over 380 nm were obtained using an excitation filter wheel (340 nm/380 nm, Chroma Technology) and UV-2A filter cube (Nikon). Images were collected every 5 sec using a 20X objective and a cooled charge-couple device (CCD) camera. SimplePCI imaging software was used for the control of filter wheel and collection of data. Tyrode’s solution (123 mM NaCl, 30 mM Glucose, 25 mM HEPES, 5 mM KCl, 2 mM CaCl_2_ and 1 mM MgCl_2_) mimicking cerebrospinal fluid was used as bath solution and the pH was adjusted to 7.4 before each experiment. Cells were perfused with the bath solution at the constant rate of 1 drop/2 sec during Ca^2+^ imaging process, and this rate was also used for the application of different drugs as described in figure legends. Triangular shaped pyramidal neurons were selected for imaging and the soma was selected as the region of interest. Sister coverslips from 2 to 3 independent cultures were used for each experiment, and pooled data was analyzed.

Ca^2+^ imaging measurements were analyzed by calculating the area under the curve (AUC). Due to the inherent variation of primary neuron culture, a small percentage of neurons displayed high Ca^2+^ transients, which obscured the drug-stimulated intracellular Ca^2+^ rise. For this reason, basal Ca^2+^ transients over 100 sec were analyzed, and those with AUC >2 (<5 %) were excluded from the final analysis.

### Western blot analysis

To determine PERK knockdown efficiency in genetic *Perk* knockout neurons, mouse cerebral cortex was isolated from day 0 *Perk-floxed Nestin-Cre* pups, and homogenized mechanically in ice-cold buffer (100 mM HEPES, 1 mM EDTA, 2 mM EGTA, 0.5 mM DTT, supplemented with 1X protease inhibitor and 1X phosphatase inhibitor cocktails; pH was adjusted to 7.0 before use) using a polypropylene pestle. Tissue lysates for western blot were prepared using RIPA buffer with 1X protease inhibitor and 1X phosphatase inhibitor cocktails. Samples were denatured by boiling in 2X Laemmli buffer for 5 min. The following primary antibodies were used in western blot analysis: monoclonal rabbit anti-PERK (Cell Signaling), monoclonal mouse anti-β-actin (GenScript).

### Synapse formation analysis

Immunocytochemical detection of the presynaptic marker Synapsin 1 and dendritic marker MAP2 was used to visualize synapses. Neurons seeded on glial-coated coverslips were fixed with 4 % paraformaldehyde, 4 % sucrose in PBS for 15 min at room temperature, permeabilized with 0.3 % Triton X-100 in PBS for 10 min, and blocked with 5 % horse serum (Invitrogen) in PBS containing 0.1 % Triton X-100 for 1 h. The cells were then incubated with the mixture of following primary antibodies overnight at 4 °C: mouse anti-Synapsin 1 (Chemicon), rabbit anti-MAP2 (Millipore). Appropriate secondary antibodies conjugated with Alexa Fluor 546 or 488 (Molecular Probes) were applied for 1 h to visualize the signals. The coverslips were mounted using the anti-fade reagent with DAPI (Molecular Probes). The fluorescent images were captured at 40X with consistent fluorescent intensities using Nikon Eclipse E1000 and SPOT 5.1 software.

To compare synapse formation density between wild-type and *Perk* knockout neurons, Synapsin 1 positive puncta in contact with MAP2 labeled dendrites within middle basal dendrites were counted, and the dendrite length was measured using ImageJ.

Additional information regarding the protocols u﻿sed in ﻿the supplemental figures is provided in (Additional file 1.

## Results

### Acute PERK inhibition impairs G_q_ protein-coupled intracellular Ca^2+^ rise in primary cortical neurons

To acutely inhibit PERK enzyme activity in primary cortical neurons, we took advantage of a highly specific inhibitor of PERK, GSK2606414 (PERKi), which acts by competing for the ATP binding domain in the catalytic site [11]. Previously we have shown that 500 nM PERKi pretreatment for 15 min sufficiently abolishes thapsigargin induced PERK activation and eIF2α phosphorylation in primary neurons [7]. In addition, it has been shown by others that 500 nM PERKi pretreatment fully inhibits PERK enzyme activity in a variety of cell types [11, 12]. Taken together, the above results suggest that 500 nM of the PERKi is effective in acute PERK inhibition.

At the gross subcellular level PERK has been shown to be expressed in both cell body and dendrites [9]. To determine if PERK is also present in the proximate region of the synapse, we examined isolated synaptoneurosomes from wild-type mouse prefrontal cortex and found that PERK was indeed present there (Additional file 2: Figure S1). Therefore PERK is available for participating in the molecular processes that govern synaptic plasticity.

**Fig. 1:**
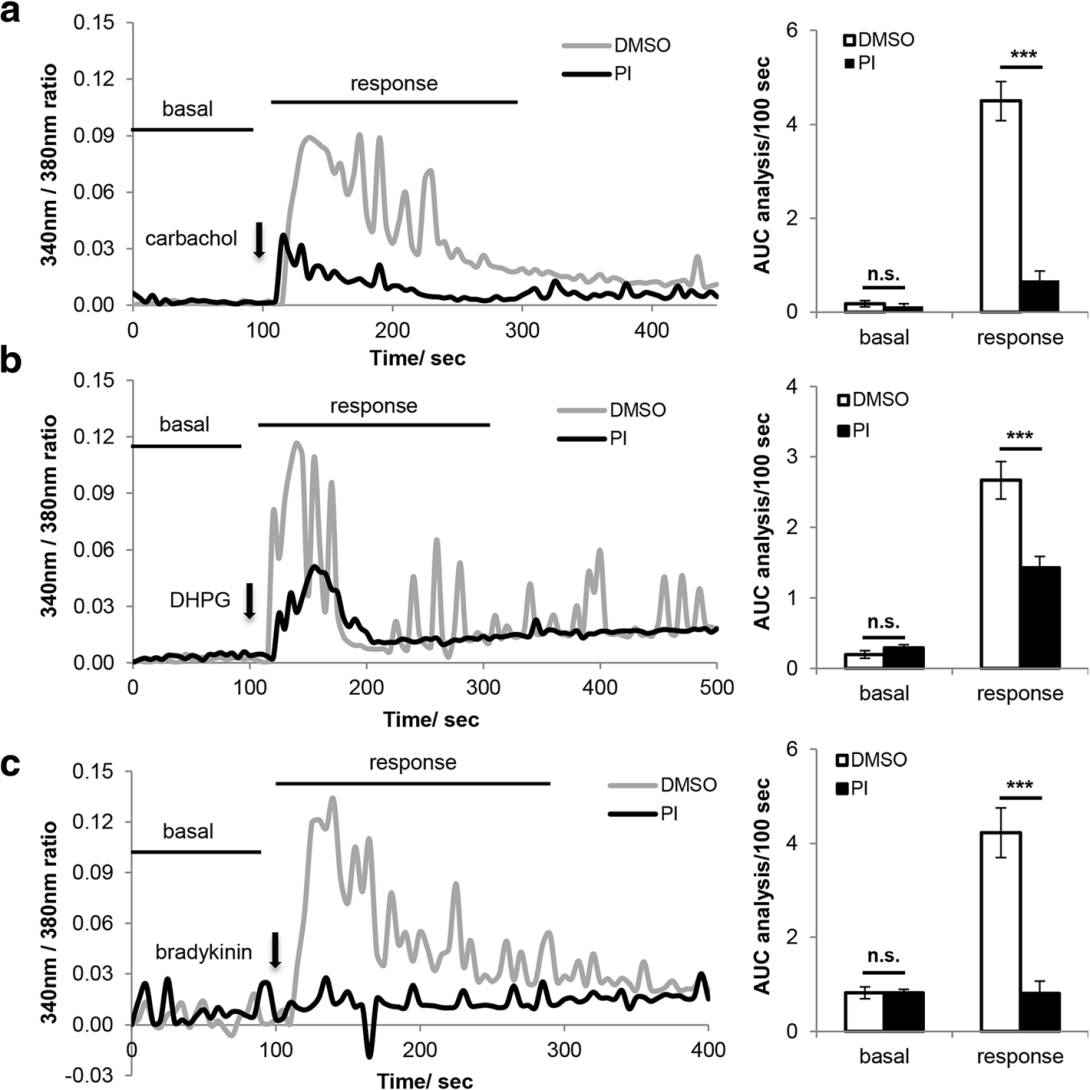
G_q_ protein-coupled intracellular Ca^2+^ ([Ca^2+^]_i_) rise is impaired by acute PERK inhibition. **a** [Ca^2+^]_i_ of primary cortical neurons in response to 250 μM carbachol treatment (DMSO *n* = 21, PI *n* = 17; ***p < 0.001, two-tailed student’s t-Test). **b** [Ca^2+^]_i_ of primary cortical neurons in response to 50 μM DHPG treatment (DMSO *n* = 36, PI *n* = 57; ***p < 0.001, two-tailed student’s t-Test). **c** [Ca^2+^]_i_ of primary cortical neurons in response to 1 μM bradykinin treatment (DMSO *n* = 25, PI *n* = 37; ***p < 0.001, two-tailed student’s t-Test). In all of the experiments above, cells were pretreated with 500 nM PERK inhibitor (PI) or DMSO for 15 min before recording. In the representative graph on the left, each Ca^2+^ trace represents the average of 6–11 neurons that were imaged from the same coverslip. Basal Ca^2+^ oscillation over 100 sec before treatment and drug-stimulated [Ca^2+^]_i_ rise over 200 sec were quantified by calculating the area under the curve (AUC). Final analysis is presented as AUC/100 sec and shown in the bar graph on the right

To examine if acute PERK inhibition impairs G_q_ protein-coupled intracellular Ca^2+^ ([Ca^2+^]_i_) rise, an intracellular response which is closely related to working memory, we used Fura-2 AM to visualize [Ca^2+^]_i_ dynamics in primary cortical neurons. We first tested if PERKi impacts cholinergic activation induced [Ca^2+^]_i_ rise by treating neurons with 250 μM carbachol, and found that 15 min 500 nM PERKi pretreatment significantly reduced carbachol-induced [Ca^2+^]_i_ rise (Fig. 1a). The acetylcholine receptor agonist, carbachol, activates both muscarinic acetylcholine and nicotinic acetylcholine receptors with the former being a G_q_ protein-coupled receptor, and the latter a Ca^2+^-permeable ionotropic receptor [13]. Thus the carbachol-induced [Ca^2+^]_i_ rise can be attributed to the stimulation of either receptor. To further examine how PERK inhibition impacts G_q_ protein-coupled intracellular Ca^2+^ signaling, we tested another G_q_ protein-coupled receptor, mGluR1, by the use of its specific agonist, DHPG. Again, acute PERK inhibition significantly suppressed 50 μM DHPG induced [Ca^2+^]_i_ rise (Fig. 1b). We also tested a third G_q_ protein-coupled receptor, the bradykinin-2 receptor, using its specific agonist, bradykinin, and found that 1 μM bradykinin induced [Ca^2+^]_i_ increase was almost abolished by PERKi treatment (Fig. 1c). That PERK inhibition is able to block cytosolic Ca^2+^ influx stimulated by three different G_q_ protein-coupled receptors strongly argues that PERKi impacts the G_q_/PLC pathway at a point downstream of receptor binding. Since PERK is present in dendrites in addition to cell body, we examined if PERK also regulates G_q_ protein-coupled Ca^2+^ rise in proximal dendrites. Indeed, acute PERK inhibition impairs DHPG-stimulated Ca^2+^ rise in proximal dendrites (Additional file 3: Figure S2).

### IP_3_-AM induced [Ca^2+^]_i_ rise is impaired by acute PERK inhibition

**Fig. 2:**
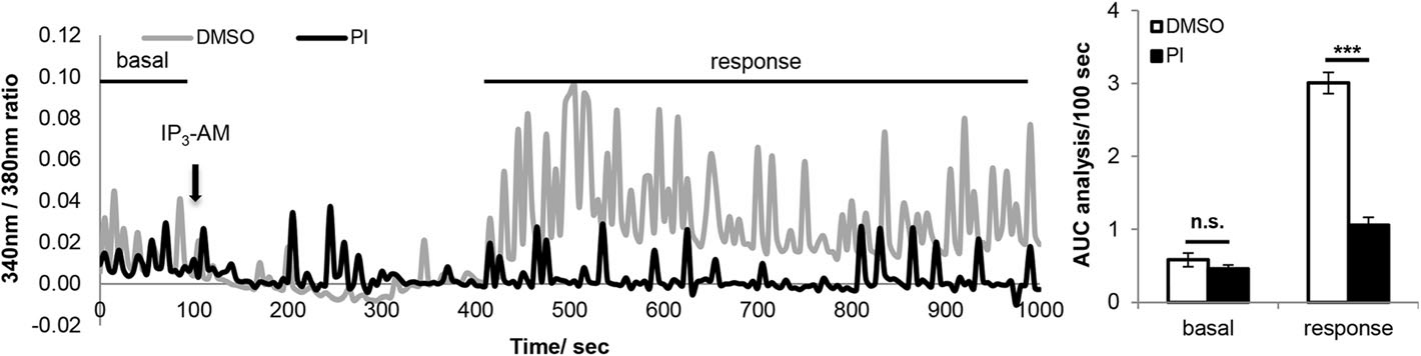
IP_3_-AM induced intracellular Ca^2+^ ([Ca^2+^]_i_) rise is impaired by acute PERK inhibition. [Ca^2+^]_i._ of primary cortical neurons in response to 1 μM IP_3_-AM treatment (DMSO *n* = 48, PI *n* = 48; *** *p* < 0.001, two-tailed student’s t-Test). Cells were pretreated with 500 nM PERK inhibitor (PI) or DMSO for 15 min before recording. In the representative graph on the left, each Ca^2+^ trace represents the average of 12–14 neurons that were imaged from the same coverslip. Basal Ca^2+^ oscillation over 100 sec before treatment and IP_3_-AM-stimulated [Ca^2+^]_i_ rise over 600 sec were quantified by calculating the area under the curve (AUC). Final analysis is presented as AUC/100 sec and shown in the bar graph on the right

Receptor-simulated activation of the G_q_/PLC pathway produces IP_3_ and increases [Ca^2+^]_i_ by induction of IP_3_ receptor mediated ER Ca^2+^ release and receptor-operated Ca^2+^ influx. To determine if PERKi affects PLC activity or a downstream Ca^2+^ channel, IP_3_-AM induced [Ca^2+^]_i_ rise was examined in PERK-inhibited neurons and DMSO controls. Cell permeable IP_3_-AM induced a delayed but sustained [Ca^2+^]_i_ rise in DMSO controls (Fig. 2). The delayed response likely reflects the time required to remove the AM moiety by cellular esterases. Acute PERK inhibition substantially suppressed IP_3_-AM induced [Ca^2+^]_i_ rise which does not require PLC activity, indicating that the likely target for PERKi is a downstream Ca^2+^ channel rather than PLC.

### Acute PERK inhibition increases IP_3_ receptor mediated ER Ca^2+^ release

**Fig. 3:**
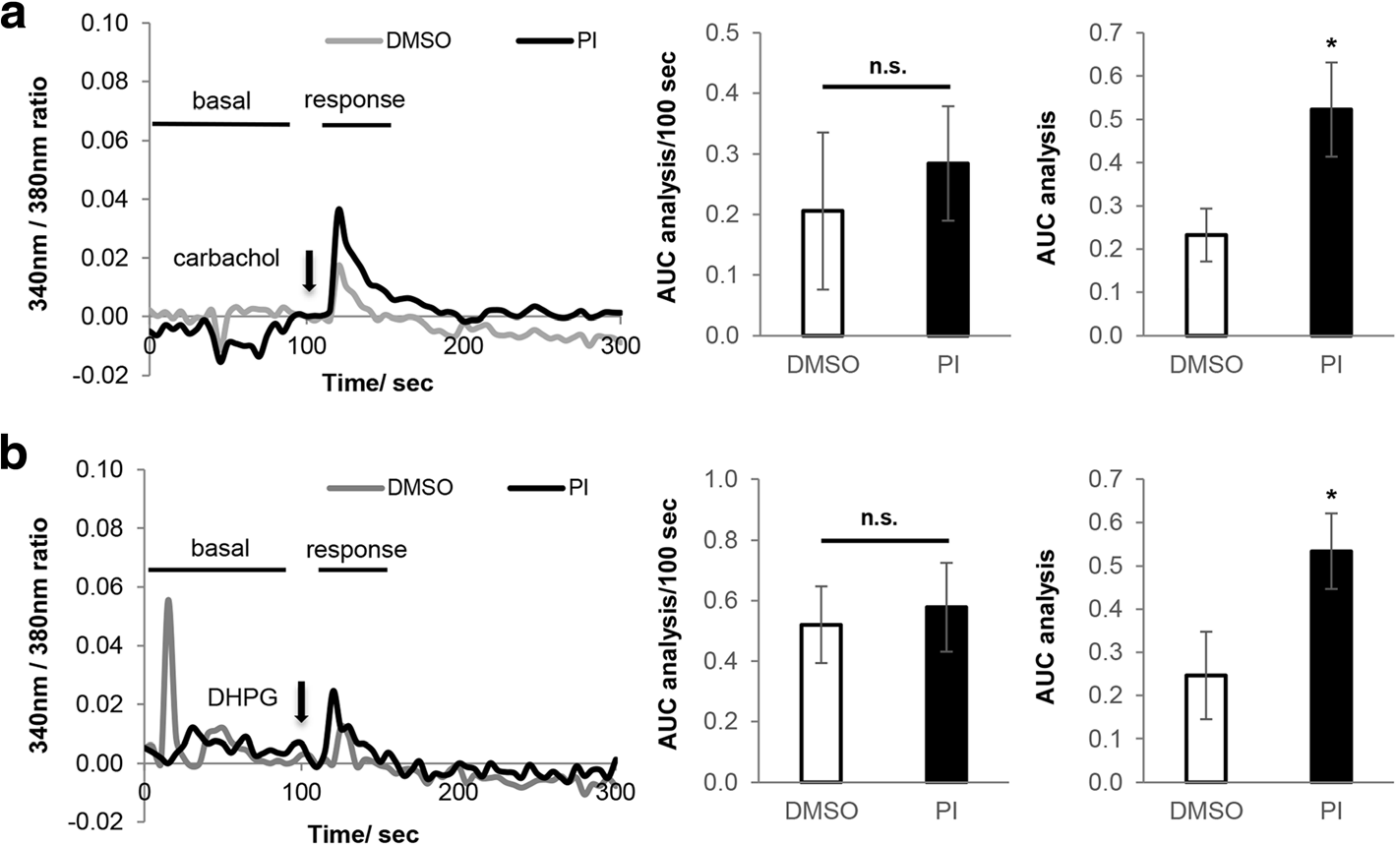
Acute PERK inhibition increases IP_3_ receptor mediated ER Ca^2+^ release. **a** [Ca^2+^]_i._ of primary cortical neurons in response to 250 μM carbachol treatment in Ca^2+^ free bath (DMSO *n* = 29, PI = 26; * *p* < 0.05, two-tailed student’s t-Test). **b** [Ca^2+^]_i._ of primary cortical neurons in response to 50 μM DHPG treatment in Ca^2+^- free bath (DMSO *n* = 33, PI = 39; * *p* < 0.05, two-tailed student’s t-Test). In both experiments, cells were pretreated with 500 nM PERK inhibitor (PI) or DMSO for 15 min before recording. Drug treatment started 100 sec after Ca^2+^- free bath perfusion. In the representative graph on the left, each Ca^2+^ trace represents the average of 8–12 neurons that were imaged from the same coverslip. Basal Ca^2+^ oscillation over 100 sec before treatment and drug-stimulated [Ca^2+^]_i_ rise over 20–30 sec were quantified by calculating the area under the curve (AUC), and shown in the middle and right bar graphs respectively

Two sources of Ca^2+^ influx contribute to G_q_ protein-coupled [Ca^2+^]_i_ increase: IP_3_R mediated ER Ca^2+^ release and receptor-operated Ca^2+^ entry (ROCE) from extracellular medium. To study PERKi’s effect on internal Ca^2+^ release, we measured [Ca^2+^]_i_ rise upon carbachol treatment in the absence of extracellular Ca^2+^, to exclude any contribution from nicotinic acetylcholine receptor or receptor-operated Ca^2+^ channel (ROCC)-dependent Ca^2+^ influx. Cells were perfused with Ca^2+^-free bath for 100 sec before stimulation with 250 μM carbachol. Carbachol treatment in Ca^2+^-free bath triggered a transient and small [Ca^2+^]_i_ increase due to Ca^2+^ release from intracellular stores, which was significantly higher in PERK-inhibited neurons (Fig. 3a). The experiment was repeated using 50 μM DHPG to stimulate mGluR1 and similar result was obtained (Fig. 3b). Taken together, these results suggest that acute PERK inhibition increases IP_3_R mediated ER Ca^2+^ release.

### Acute PERK inhibition impairs receptor-operated Ca^2+^ entry, but not store-operated Ca^2+^ entry

**Fig. 4:**
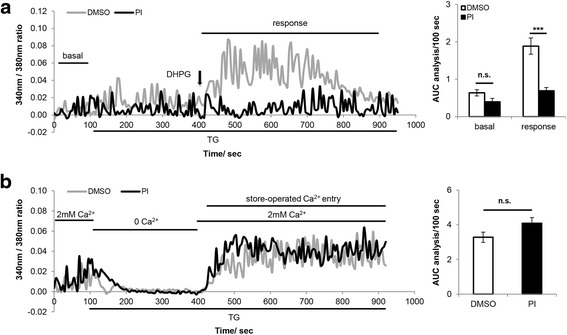
Acute PERK inhibition impairs receptor-operated Ca^2+^ entry, but not store-operated Ca^2+^ entry. **a** [Ca^2+^]_i._ of thapsigargin (TG) pretreated primary cortical neurons in response to 50 μM DHPG treatment. Cells were pretreated with 500 nM PERK inhibitor (PI) or DMSO for 15 min before recording, and perfused with 1 μM TG for 300 sec before 50 μM DHPG treatment. In the representative graph on the left, each Ca^2+^ trace represents the average of 8–9 neurons that were imaged from the same coverslip. Basal Ca^2+^ oscillation over 100 sec before treatment and DHPG-stimulated [Ca^2+^]_i_ rise over 500 sec were quantified by calculating the area under the curve (AUC). Final analysis is presented as AUC/100 sec and shown in the bar graph on the right (DMSO *n* = 37, PI *n* = 35; *** *p* < 0.001, two-tailed student’s t-Test). **b** Store-operated Ca^2+^ entry in primary cortical neurons. Cells were pretreated with 500 nM PI or DMSO for 15 min before recording, and perfused with 1 μM TG in Ca^2+^- free bath for 300 sec before reintroduction of 2 mM Ca^2+^. In the representative graph on the left, each Ca^2+^ trace represents the average of 9–12 neurons that were imaged from the same coverslip. Store-operated Ca^2+^ entry over 500 sec was quantified by calculating the area under the curve (AUC). Final analysis is presented as AUC/100 sec and shown in the bar graph on the right (DMSO *n* = 45, PI *n* = 36; n.s. not significant, two-tailed student’s t-Test)

Our observation that acute PERK inhibition impairs G_q_ protein-coupled [Ca^2+^]_i_ mobilization and increases IP_3_R-dependent ER Ca^2+^ release suggests that ROCE is impaired as a result of PERKi treatment. To test this hypothesis, DHPG stimulated ROCE was examined in PERK-inhibited neurons and DMSO controls after ER Ca^2+^ depletion by the use of a SERCA pump inhibitor, thapsigargin [14]. The pretreatment with thapsigargin caused a rapid and irreversible depletion of ER Ca^2+^. Thus upon DHPG stimulation, the rise of [Ca^2+^]_i_ in ER Ca^2+^ depleted-neurons was largely contributed by ROCC-dependent extracellular Ca^2+^ influx. PERKi treatment significantly reduced DHPG induced [Ca^2+^]_i_ rise in ER Ca^2+^ depleted-neurons, indicating that ROCC-dependent extracellular Ca^2+^ influx is impaired upon PERK inhibition (Fig. 4a).

Store-operated Ca^2+^ entry (SOCE) refers to cytosol Ca^2+^ influx mediated by cell membrane Ca^2+^ channels triggered by ER Ca^2+^ store depletion. Since ROCE and SOCE are two closely related processes, and store depletion is an integral component of ROCE, we next examined PERKi’s effect on SOCE in primary cortical neurons. As shown in Fig. 4a, in neurons perfused with Ca^2+^-containing bath, thapsigargin treatment only elicited a transient [Ca^2+^]_i_ rise, which is the result of the combined effect of thapsigargin-induced ER Ca^2+^ release and SOCE, suggesting that thapsigargin stimulation alone did not significantly induce SOCE in primary neurons. To maximally activate SOCE, we followed a “Ca^2+^ re-addition” protocol [15], where cells were treated with 1 μM thapsigargin in Ca^2+^-free bath for 300 sec to fully deplete ER Ca^2+^ and activate store-operated Ca^2+^ channels (SOCC). Subsequent reintroduction of 2 mM Ca^2+^ into the bath elicited a sustained [Ca^2+^]_i_ elevation, reflecting SOCC mediated Ca^2+^ influx. No difference was observed between PERK-inhibited neurons and DMSO controls (Fig. 4b), suggesting that acute PERK inhibition does not affect SOCE. Previous studies have shown that thapsigargin induced SOCE in pyramidal neurons is L-type voltage-gated Ca^2+^ channel (VGCC)-independent [16], thus L-type VGCC inhibitor was not included in the bath.

### G_q_ protein-coupled [Ca^2+^]_i_ rise is impaired in genetic *Perk* knockout primary cortical neurons

**Fig. 5:**
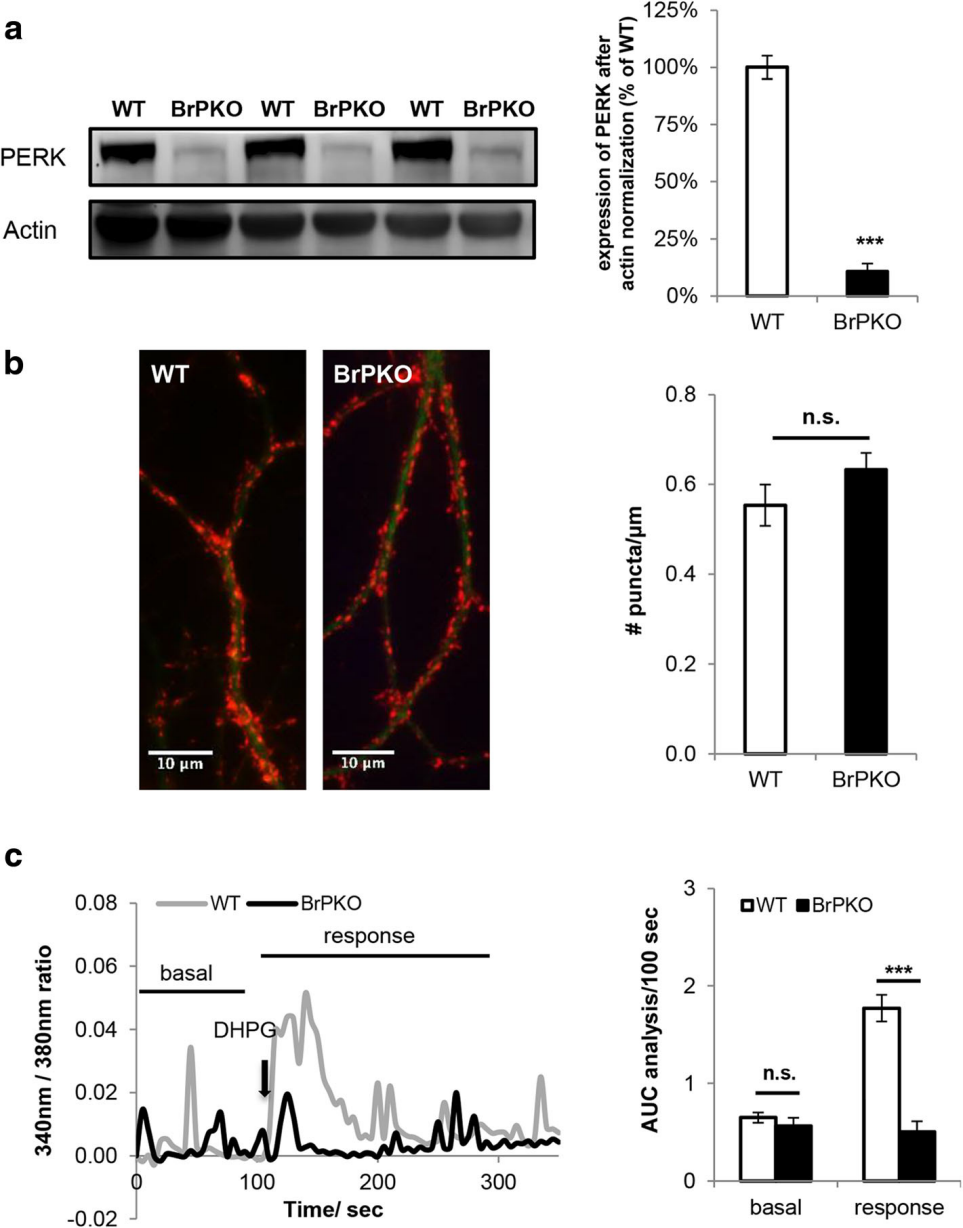
G_q_ protein-coupled intracellular Ca^2+^ ([Ca^2+^]_i_) mobilization is impaired in genetic *Perk* knockout primary cortical neurons. **a** Western blot analysis confirmed almost complete knockdown of PERK in the cerebral cortex of *BrPKO* mice at postnatal day 0 (*BrPKO*: *Nestin-Cre Perk*-floxed; *** *p* < 0.001, two-tailed student’s t-Test). **b** No difference in synapse density was observed between WT and *BrPKO* primary cortical neurons. Representative image on the left shows the immunofluorescent staining of Synapsin 1(red) and MAP2 (green) in primary cortical neurons. Synapse density quantification in the bar graph on the right represents pooled data from 3 mice per genotype (5 neurons were randomly picked for synapse density quantification per animal, *n* = 15 for each genotype; WT and *BrPKO* neurons were cultured from the pups in the same litter; n.s. not significant, two-tailed student’s t-Test). **c** DHPG stimulated [Ca^2+^]_i_ rise is impaired in genetic *Perk* KO primary cortical neurons. In the representative graph on the left, each Ca^2+^ trace represents the average of 8–10 neurons that were imaged from the same coverslip. Basal Ca^2+^ oscillation over 100 sec before treatment and DHPG-stimulated [Ca^2+^]_i_ rise over 200 sec were quantified by calculating the area under the curve (AUC). Final analysis is presented as AUC/100 sec and shown in the bar graph on the right (WT *n* = 44, *BrPKO n* = 34; *** *p* < 0.001, two-tailed student’s t-Test)

To investigate if the impaired G_q_ protein-coupled [Ca^2+^]_i_ mobilization could be mimicked by genetic ablation of *Perk*, primary cortical neurons from brain-specific *Perk* KO (*BrPKO*) mice were examined. *BrPKO* mice were generated by crossing *Perk*-floxed mice [17] with the transgenic *Nestin-Cre* mice strain [18], which enables widespread deletion of the *loxP*-flanked *Perk* gene sequence in neurons and glial cells during embryonic stage [19, 20]. Western blot analysis confirmed almost complete knockdown of PERK in the cerebral cortex of *BrPKO* mice at postnatal day 0 (Fig. 5a). With the concern that knockdown of PERK may affect neuronal differentiation and synapse formation in vitro, synapse density was examined in *BrPKO* and wild-type primary cortical neurons by double immunofluorescence staining of the presynaptic marker Synapsin 1 and the dendritic marker MAP2 prior to examining their G_q_ protein-coupled [Ca^2+^]_i_ rise. No significant difference was observed in synapse density between genotypes (Fig. 5b). To determine if G_q_ protein-coupled [Ca^2+^]_i_ mobilization is impaired in *BrPKO* primary cortical neurons, mGluR1 agonist DHPG was applied, and significantly smaller DHPG-stimulated [Ca^2+^]_i_ rise was observed in *BrPKO* neurons (Fig. 5c), which is consistent with the pharmacological PERK inhibition results.

## Discussion

Although earlier studies have demonstrated that PERK plays an important role in regulating cognitive functions including behavior flexibility [8] and mGluR1-dependent long-term depression [9], the underlying mechanisms remain unknown. Previously we showed that PERK regulates Ca^2+^ dynamics in electrically excitable pancreatic β cells [10], and modulates Ca^2+^ dynamics-dependent working memory [7], suggesting that PERK may regulate Ca^2+^ dynamics in neurons. Neuronal cytosolic Ca^2+^ rise is contributed by two major Ca^2+^ sources: internal Ca^2+^ release mediated by ER-resident IP_3_R or Ryanodine receptor, and external Ca^2+^ influx mediated by voltage-dependent Ca^2+^ channel, ionotropic glutamate receptor, nicotinic acetylcholine receptor, or TRPCs [21]. PERK’s subcellular localization in the soma, dendrites and synaptoneurosomes suggests the possibility that it plays multiple roles in Ca^2+^ channel regulation. Moreover, its localization within ER membrane and primary spatial expression in soma and dendrites are functionally important for its regulation of ER-resident IP_3_R, and potential regulation of TRPCs, which are localized mainly in soma and dendrites [22–24].

In this study, we investigated the role of PERK in G_q_ protein-coupled [Ca^2+^]_i_ mobilization in primary cortical neurons, and identified it as a negative regulator of IP_3_R-dependent ER Ca^2+^ release and a positive regulator of receptor-operated Ca^2+^ entry. Our finding that inhibition of PERK alters Ca^2+^ dynamics within a few minutes after inhibitor application is inconsistent with the hypothesis that these effects are mediated by changes in protein translation. Moreover, it is unlikely that these observations are due to off-target effects because genetic ablation of *Perk* mimicked the impaired G_q_ protein-coupled [Ca^2+^]_i_ mobilization observed in pharmacologically PERK-inhibited neurons.

How then does PERK regulate these processes? We speculate that PERK’s regulation of IP_3_R-dependent ER Ca^2+^ release is mediated by its regulation of calcineurin, a Ca^2+^/calmodulin-dependent protein phosphatase that negatively regulates IP_3_R [25, 26]. PERK and calcineurin have been shown to physically interact, which impacts their individual enzymatic activities [27]. Moreover, in pancreatic insulin-secreting β-cells, PERK positively regulates calcineurin activity and calcineurin is a downstream mediator of PERK’s action on Ca^2+^-dependent insulin secretion [10]. These results led us to speculate that PERK might negatively regulate IP_3_R activity through its positive regulation of calcineurin in pyramidal neurons.

For G_q_/PLC coupled ROCE, the family of TRPC channels form nonselective receptor-operated Ca^2+^ channels [28]. A number of intracellular signals generated downstream of G_q_/PLC pathway have been shown to activate TRPCs, which include increased PLC activity, generation of DAG and internal Ca^2+^ store depletion [28]. Among them, DAG is the only identified second messenger that directly gates TRPC activity. DAG has been shown to activate TRPC3/6/7 channels [29, 30] while inhibiting TRPC5 channel activity [31]. Since PERK has an intrinsic DAG kinase activity of converting DAG into phosphatidic acid [32], it is possible that PERK regulates TRPC activity by modulating intramembrane DAG levels. In addition, it is also possible that PERK regulates ROCE via its interaction with calcineurin. In neuronal PC12D cells, it has been shown that calcineurin is recruited to the TRPC6 centered multiprotein complex induced by M1 mAChR activation, and it is essential for TRPC6 dephosphorylation and M1 mAChR dissociation from the complex, suggesting that calcineurin might play a regulatory role in receptor-operated TRPC6 activation [33].

Receptor-operated and stored-operated Ca^2+^ entries are closely related: store depletion is an integral component of ROCE, and TRPCs have been suggested to be the Ca^2+^ channels involved in both processes. Although almost all of the TRPCs can be activated by store depletion [34–41], there is accumulating evidence suggesting that the regulation of TRPC3/6/7 [29, 30, 42] and TRPC4/5 [43, 44] activities can also be store depletion-independent. Our observation that acute PERK inhibition impairs ROCE but not SOCE suggests that PERK’s regulation of ROCE might be independent of internal Ca^2+^ release.

Does PERK’s regulation of G_q_ protein-coupled [Ca^2+^]_i_ mobilization play any physiological role in cognitive function? Previously we have observed significant working memory impairment in forebrain-specific *Perk* KO mice [7], and we speculate that PERK regulates working memory via its modulation of G_q_ protein-coupled Ca^2+^ dynamics in pyramidal neurons. Intracellular signaling pathways initiated by muscarinic acetylcholine and metabotropic glutamate receptors are critical for working memory, since blockage of either receptor impairs working memory in animals [45–48], and activation of either receptor is sufficient to induce the Ca^2+^-activated nonselective cationic current (I_CAN_) [4, 5] , which is essential for working memory. G_q_ protein-coupled [Ca^2+^]_i_ mobilization regulates working memory in several ways. First, the induced [Ca^2+^]_i_ rise is known to activate several Ca^2+^-dependent protein enzymes, including the phosphatase calcineurin, and the kinases CaMKII and PKC, all of which have been shown to regulate working memory capacity [49]. Secondly, the I_CAN_, which is identified as the ionic mechanism underlying neuronal persistent firing [4], is G_q_ protein and Ca^2+^-dependent [5]. Finally, G_q_ protein-coupled [Ca^2+^]_i_ rise has direct effects on intrinsic neuronal excitability. It has been demonstrated that pharmacological activation of mGluR1 in prefrontal cortex pyramidal neurons triggers a biphasic electrical response-SK channel-dependent neuronal hyperpolarization followed by TRPC-dependent neuronal depolarization, and the amplitude of both are regulated by the extent of [Ca^2+^]_i_ rise [50, 51]. Taken together, we speculate that PERK may regulate working memory by modulating G_q_ protein-coupled [Ca^2+^]_i_ mobilization in pyramidal neurons.

Considering PERK’s role in eIF2α-dependent protein synthesis and translational control, it has been hypothesized that PERK’s regulation over memory flexibility and mGluR1-dependent long-term depression is eIF2α-dependent [8, 9]. However, genetic reduction of eIF2α phosphorylation by single allele phosphorylation site mutation of eIF2α [52], or knockdown of other eIF2α kinases GCN2 [53] and PKR [54], lowers the threshold for late phase long-term potentiation and facilitates long-term memory storage, a phenotype that is absent in forebrain-specific *Perk* knockout mice [8, 9]. Thus, it is very likely that PERK imparts additional regulation on cognition that is eIF2α-independent. This study’s discovery of PERK-dependent regulation of G_q_ protein-coupled Ca^2+^ dynamics in primary cortical neurons, together with the earlier finding that PERK regulates Ca^2+^ dynamics-dependent working memory [7], supports the above hypothesis. Further studies are required to elucidate the specific pathways that underlie PERK’s regulation of intracellular Ca^2+^ dynamics.

**Fig. 6:**
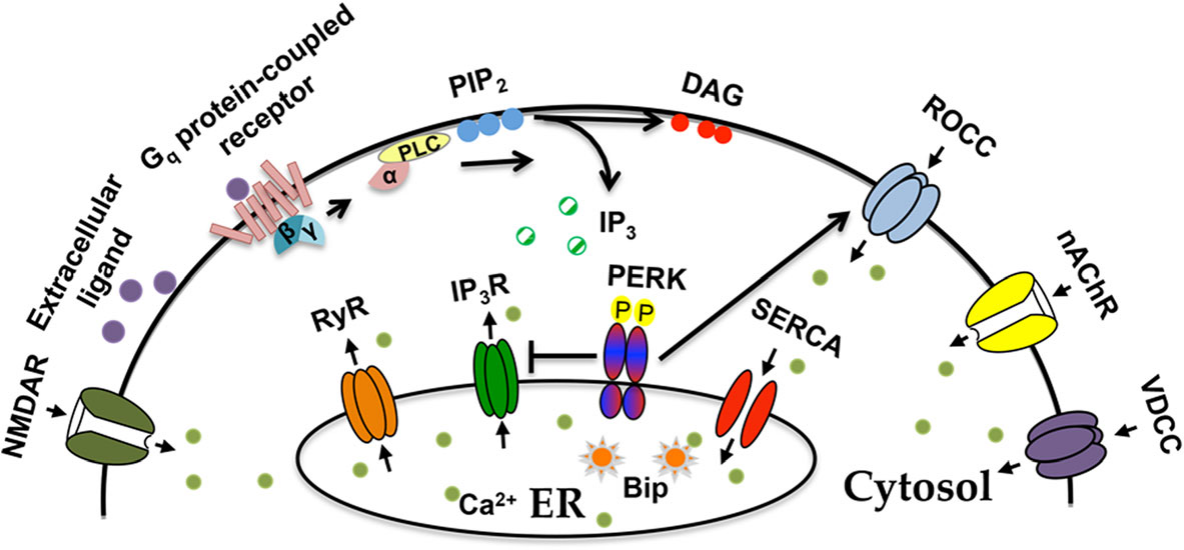
Proposed model for PERK’s regulation of G_q_ protein-coupled Ca^2+^ dynamics in pyramidal neurons. Upon extracellular ligand binding, G_q_ protein-coupled receptor is activated, which subsequently activates G_q_/PLC. Activated PLC hydrolyzes PIP_2_ into IP_3_ and DAG. Increased cytosol IP_3_ induces ER Ca^2+^ depletion by binding with ER-resident IP_3_R, which may activate PERK due to Ca^2+^ dissociation from its regulatory domain in the ER. Activated PERK may then restore ER Ca^2+^ level by inhibiting IP_3_R mediated ER Ca^2+^ release and activating receptor-operated Ca^2+^ entry

As an eIF2α kinase, how did PERK evolve to be a modulator of G_q_ protein-coupled Ca^2+^ dynamics in pyramidal neurons? We speculate that during early vertebrate evolution, PERK first played an eIF2α-dependent role in CNS. Given its localization on the ER, which is the major organelle for intracellular Ca^2+^ storage, and its regulation by ER/cytosolic Ca^2+^[10, 55], the constant interaction with Ca^2+^ may have provided PERK the opportunity to evolve an additional function to regulate intracellular Ca^2+^ dynamics through mechanism independent of eIF2a and protein translation. The fact that PERK is activated by ER Ca^2+^ depletion [55], and the discoveries of PERK being a negative regulator of IP_3_R and a positive regulator of ROCC shown herein, fit well into this hypothesis: when ER Ca^2+^ stores are depleted under physiological responses such as activation of G_q_ protein-coupled receptor, PERK is activated due to Ca^2+^ dissociation from its regulatory domain in the ER, and it subsequently replenishes ER Ca^2+^ by inhibiting IP_3_R mediated ER Ca^2+^ release and activating ROCE (Fig. 6).

## Additional files

Additional file 1: Materials and methods for supplemental figures. (DOCX 17 kb) Additional file 2: Figure S1. PERK is expressed in synaptoneurosome. A. Western blot analysis confirmed that the synaptoneurosome fraction is enriched of presynaptic marker synaptotagmin, postsynaptic marker NMDAR 2B, and clear of a nuclear marker CREB-1. Representative western blot on the left shows the expression of fraction markers in the homogenate and synaptoneurosome isolated from mice prefrontal cortex. (H: homogenate; S: synaptoneurosome). The protein quantification on the right represents pooled data from both genotypes (*n* = 6 for each genotype; * *p* < 0.05; *** *p* < 0.001; two-tailed student’s t-Test). B. PERK signal in synaptoneurosome is not due to rough ER contamination from soma. The ratio of PERK’s expression in synaptoneurosome over homogenate fraction is compared to that of a rough ER marker, Ribophorin 1. The significant higher ratio of PERK suggests that the detection of PERK signal in synaptoneurosome is not due to rough ER contamination from soma. Representative western blot on the left shows the expression of PERK and Ribophorin 1 in the homogenate and synaptoneurosomes collected from wild-type mice’s prefrontal cortex. The quantification on the right represents the ratio of each protein’s expression in synaptoneurosome over homogenate fraction after actin normalization (* *p* < 0.05, two-tailed student’s t-Test). (TIF 272 kb) Additional file 3: Figure S2. DHPG induced Ca^2+^ rise in proximal dendrites is impaired by acute PERK inhibition. Ca^2+^ level in the proximal dendrites of primary cortical neurons in response to 50 μM DHPG treatment. (DMSO *n* = 18, PI *n* = 16; ** *p* < 0.01, two-tailed student’s t-Test). Cells were pretreated with 500 nM PERK inhibitor (PI) or DMSO for 15 min before recording. In the representative graph on the left, each Ca^2+^ trace represents the average of 6 proximal dendrites from 6 individual neurons that were imaged from the same coverslip. Basal Ca^2+^ oscillation over 100 sec before treatment and DHPG-stimulated Ca^2+^ rise over 200 sec were quantified by calculating the area under the curve (AUC). Final analysis is presented as AUC/100 sec and shown in the bar graph on the right. (TIF 174 kb)

## Abbreviations

[Ca^2+^]_i_: Intracellular Ca^2+^
*BrPKO*: Brain-specific *Perk* knockout
DAG: Diacylglycerol
I_CAN_: Ca^2+^-activated nonselective cationic current
IP_3_: Inositol 1,4,5-triphosphate
IP_3_R: Inositol-1,4,5-triphosphate receptor
mAChR: Muscarinic acetylcholine receptor
mGluR1: Group 1 metabotropic glutamate receptor
PERKi: PERK inhibitor
PIP_2_: Phosphatidylinositol 4,5-bisphosphate
PLC: Phospholipase C
ROCC: Receptor-operated Ca^2+^ channel
ROCE: Receptor-operated Ca^2+^ entry
SOCC: Store-operated Ca^2+^ channel
SOCE: Store-operated Ca^2+^ entry
VGCC: Voltage-gated Ca^2+^ channel
