# Supplementary material for: PERK regulates Gq protein-coupled intracellular Ca2+ dynamics in primary cortical neurons
Source: Mol Brain. 2016 Oct 1;9:87. doi: 10.1186/s13041-016-0268-5 (PMC5045583; doi:10.1186/s13041-016-0268-5)
Supplement: Additional file 1: — Materials and methods for supplemental figures. (DOCX 17 kb) [file 13041_2016_268_MOESM1_ESM.docx]

**Materials and Methods for Supplementary Figure**

**Synaptoneurosome isolation and antibodies used for western blot analysis**

Synaptoneurosomes were isolated as previously described(1). Briefly, the prefrontal cortex was isolated from mouse brain and homogenized mechanically in 1.1 ml ice-cold synaptoneurosome buffer (100mM HEPES, 1mM EDTA, 2mM EGTA, 0.5mM DTT, pH 7.0, with 1X protease inhibitor and 1X phosphatase inhibitor cocktails) using a polypropylene pestle. 100ul of the homogenate was set aside for western blot analysis and the rest was diluted with 7ml ice-cold synaptoneurosome buffer and gently sonicated. The sonicated homogenate was then filtered through two layers of pre-wetted 100μm pore nylon net filters followed by filtration through one layer of pre-wetted 5μm pore hydrophilic membrane. The resulting filtrate was centrifuged at 1000*Xg* for 10 min and the pellet corresponding to synaptoneurosomes was collected.

The following primary antibodies were used in western blot analysis: rabbit anti-PERK (Cell Signaling), mouse anti-β-actin (GenScript), mouse anti-synaptotagmin (gift from Dr. Yingwei Mao, Penn State University), rabbit anti-NMDAR 2B (Santa Cruz), rabbit anti-CREB-1 (Santa Cruz), and goat anti-Ribophorin 1 (Santa Cruz).

**Ca^2+^ measurements in proximal dendrites**

To measure proximal dendrites Ca^2+^ levels, Fura-2 AM was loaded at 37 C° for 30 min to increase the probe signal in the dendrites. Other procedures were performed in the same way as described in the intracellular Ca^2+^ measurements.

**Reference**

1. Villasana, L. E., Klann, E., and Tejada-Simon, M. V. (2006) Rapid isolation of synaptoneurosomes and postsynaptic densities from adult mouse hippocampus. *Journal of neuroscience methods* **158**, 30-36
