# Supplementary figures and images for: PERK regulates Gq protein-coupled intracellular Ca2+ dynamics in primary cortical neurons
Source: Mol Brain. 2016 Oct 1;9:87. doi: 10.1186/s13041-016-0268-5 (PMC5045583; doi:10.1186/s13041-016-0268-5)

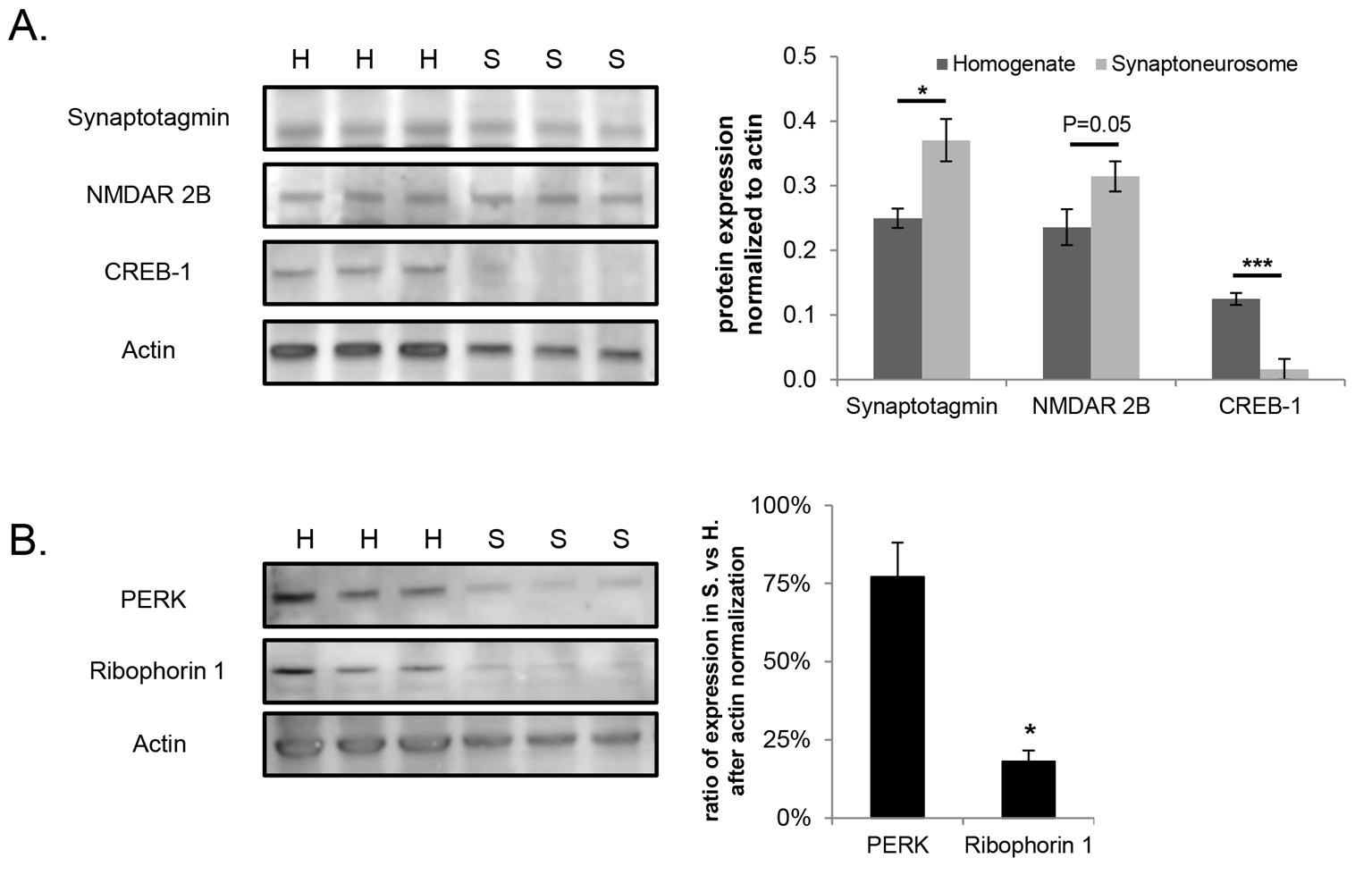

Supplement: Additional file 2: Figure S1. — PERK is expressed in synaptoneurosome. A. Western blot analysis confirmed that the synaptoneurosome fraction is enriched of presynaptic marker synaptotagmin, postsynaptic marker NMDAR 2B, and clear of a nuclear marker CREB-1. Representative western blot on the left shows the expression of fraction markers in the homogenate and synaptoneurosome isolated from mice prefrontal cortex. (H: homogenate; S: synaptoneurosome). The protein quantification on the right represents pooled data from both genotypes (n = 6 for each genotype; * p < 0.05; *** p < 0.001; two-tailed student’s t-Test). B. PERK signal in synaptoneurosome is not due to rough ER contamination from soma. The ratio of PERK’s expression in synaptoneurosome over homogenate fraction is compared to that of a rough ER marker, Ribophorin 1. The significant higher ratio of PERK suggests that the detection of PERK signal in synaptoneurosome is not due to rough ER contamination from soma. Representative western blot on the left shows the expression of PERK and Ribophorin 1 in the homogenate and synaptoneurosomes collected from wild-type mice’s prefrontal cortex. The quantification on the right represents the ratio of each protein’s expression in synaptoneurosome over homogenate fraction after actin normalization (* p < 0.05, two-tailed student’s t-Test). (TIF 272 kb) [file 13041_2016_268_MOESM2_ESM.tif]

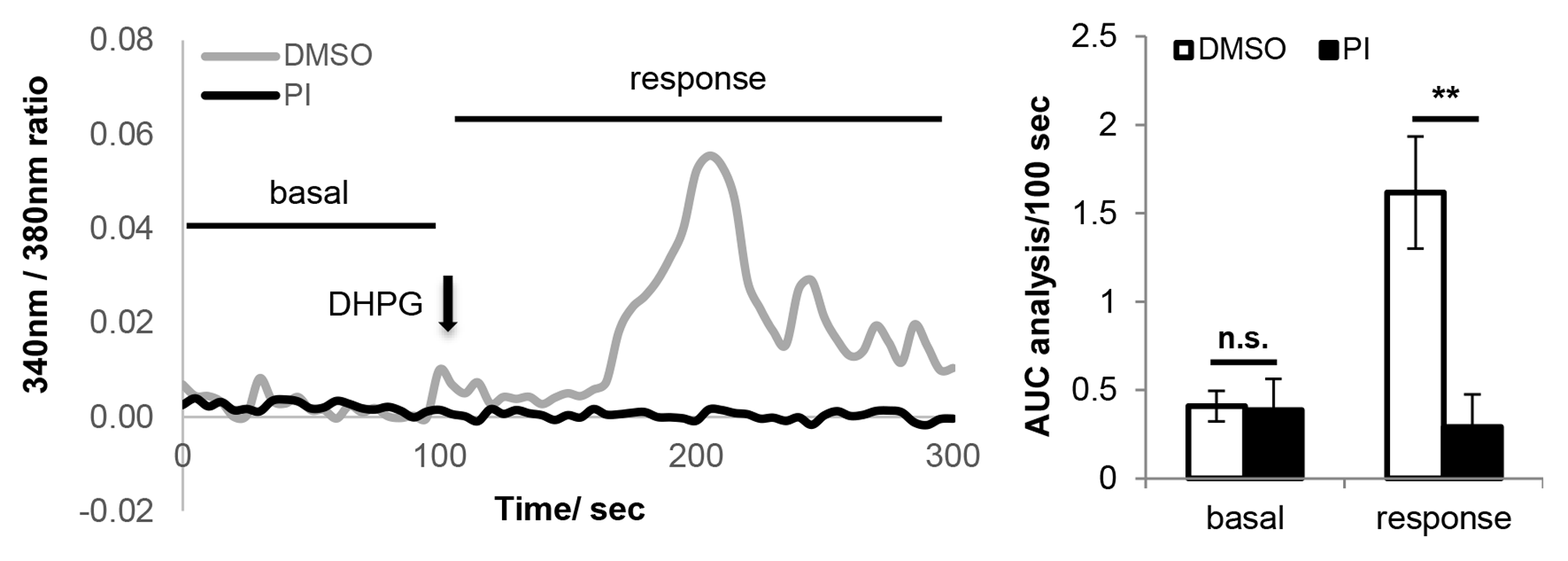

Supplement: Additional file 3: Figure S2. — DHPG induced Ca2+ rise in proximal dendrites is impaired by acute PERK inhibition. Ca2+ level in the proximal dendrites of primary cortical neurons in response to 50 μM DHPG treatment. (DMSO n = 18, PI n = 16; ** p < 0.01, two-tailed student’s t-Test). Cells were pretreated with 500 nM PERK inhibitor (PI) or DMSO for 15 min before recording. In the representative graph on the left, each Ca2+ trace represents the average of 6 proximal dendrites from 6 individual neurons that were imaged from the same coverslip. Basal Ca2+ oscillation over 100 sec before treatment and DHPG-stimulated Ca2+ rise over 200 sec were quantified by calculating the area under the curve (AUC). Final analysis is presented as AUC/100 sec and shown in the bar graph on the right. (TIF 174 kb) [file 13041_2016_268_MOESM3_ESM.tif]
